# PTEN expression is upregulated by a RNA-binding protein RBM38 via enhancing its mRNA stability in breast cancer

**DOI:** 10.1186/s13046-017-0620-3

**Published:** 2017-10-19

**Authors:** Xu-Jie Zhou, Jing Wu, Liang Shi, Xiao-Xia Li, Lei Zhu, Xi Sun, Jia-Yi Qian, Ying Wang, Ji-Fu Wei, Qiang Ding

**Affiliations:** 1 Jiangsu Breast Disease Center, the First Affiliated Hospital with Nanjing Medical University, 300 Guangzhou Road, Nanjing, 210029 China; 2 Department of Thyroid Surgery, the First Affiliated Hospital with Nanjing Medical University, 300 Guangzhou Road, Nanjing, 210029 China; 3 Research Division of Clinical Pharmacology, the First Affiliated Hospital with Nanjing Medical University, 300 Guangzhou Road, Nanjing, 210029 China; 4 Breast Disease Center, the First Affiliated Hospital with Nanjing Medical University, 300 Guangzhou Road, Nanjing, 210029 China

**Keywords:** Breast cancer, RBM38, PTEN, mRNA stability, Growth suppression

## Abstract

**Background:**

PTEN (phosphatase and tensin homolog gene on chromosome 10), a well-characterized tumor suppressor, is a key regulator of the phosphatidylinositol-3-kinase (PI3K)/AKT pathway involved in cell survival, metastasis and cell renewal. PTEN expression is closely related to the phenotype, prognosis and drug selection in breast cancer. It is mainly regulated by transcriptional and post-transcriptional modifications. RNA binding motif protein 38 (RBM38), an RNA-binding protein (RBP) and a target of P53 family, plays a crucial role in the regulation of cellular processing, especially in post-transcription regulation and gene transcription. In this study, we investigated a new post-transcription regulation mechanism of PTEN expression by RBM38 in breast cancer.

**Methods:**

Immunohistochemistry, lentivirus transfections, Western blotting analysis, qRT-PCR and ELISA were used to conduct the relation between RBM38 and PTEN. RNA immunoprecipitation, RNA electrophoretic mobility shift and dual-luciferase reporter assays were employed to identify the direct binding sites of RBM38 with PTEN transcript. Colony formation assay was conducted to confirm the function of PTEN in RBM38-induced growth suppression.

**Results:**

PTEN expression was positively associated with the expression of RBM38 in breast cancer tissues and breast cancer cells. Moreover, RBM38 stabilized PTEN transcript to enhance PTEN expression via binding to multiple AU/U- rich elements (AREs) in 3′-untranslated region (3′-UTR) of PTEN transcript. Additionally, specific inhibitors of PTEN activity and small interfering (siRNA) of PTEN expression inhibited RBM38-mediated suppression of proliferation, which implied that RBM38 acted as a tumor suppressor partly by enhancing PTEN expression.

**Conclusion:**

The present study revealed a new PTEN regulating mechanism that PTEN was positively regulated by RBM38 via stabilizing its transcript stability, which in turn alleviated RBM38-mediated growth suppression.

**Electronic supplementary material:**

The online version of this article (10.1186/s13046-017-0620-3) contains supplementary material, which is available to authorized users.

## Background

Over the past few decades, breast cancer is the most commonly diagnosed cancer and remains the leading cause of morbidity and mortality in the female population [1, 2]. It motivates broad public interests and overwhelming researches have been carried out to explore the pathogenesis, including the genes involved in regulating breast cancer growth and metastasis.

PTEN (phosphatase and tensin homolog deleted on chromosome 10), also known as TEP1 (TGFβ-regulated and epithelial cell-enriched phosphatase) or MMAC1 (mutated in multiple advanced cancers), localized on chromosome 10q23, is a well-known tumor suppressor gene [3]. PTEN is a dual phosphatase with activity on lipids and proteins which can dephosphorylate phosphatidylinositol (3,4,5)-trisphosphate (PIP_3_) to phosphatidylinositol (4,5)-trisphosphate (PIP_2_) and antagonize the PI3K/Akt oncogenic pathway to play a particularly important role in the regulation of various normal cell processes, including regulation of proliferation and survival, cell motility and migration, angiogenesis, and induction of cell-cycle checkpoints in response to DNA damage [4–6]. Recently, PTEN was found to be a positive regulator of energy expenditure, and negative regulator of nutrient storage [7]. In contrast to p53,a rapidly degraded protein [8], PTEN appears to be a relatively stable protein. Subtle changes in PTEN dose determine cancer susceptibility and contribute to tumor progression [9]. In breast tumors, PTEN is a vital prognostic factor and reduced PTEN expression was associated with aggressive phenotype and poor outcome for patients with this disease [10, 11]. It was found that the PTEN expression level affected the incidents of breast cancer in mouse model [9]. In clinical samples, loss of PTEN expression was correlated with disease-related death and lymph node metastasis [11]. Furthermore, PTEN was also involved in considerable target therapies in breast cancer, including endocrine therapy and humane epidermal growth factor receptor-2 (HER2) target therapy. The reduction of PTEN could activate the PI3K signaling pathway and generate a gene signature associated with luminal B subtype of breast cancer to cause endocrine resistance in estrogen receptor (ER) positive breast cancer. While activating of PTEN is a novel mechanism underlying trastuzumab’s antitumor activity and loss of PTEN predicts trastuzumab resistance in patients [12, 13]. All these findings implied that the expression of PTEN was closely related to the phenotype, prognosis and drug selection in breast cancer.

PTEN expression was precisely regulated on its transcriptional and post-transcriptional modifications, including epigenetic silencing, microRNA (miRNA) regulation, abnormal localization of PTEN, PTEN-interacting proteins regulation. Furthermore, the lipid phosphatase activity of PTEN could also be controlled post-transcriptionally via ubiquitination, inhibitory phosphorylation or oxidation [14]. Numerous miRNAs, such as miR-222 and miR-10b, were demonstrated to promote tumorigenesis by downregulating PTEN expression [15–18]. Despite of the considerable evidences for PTEN regulation, the exact functional consequences and mechanisms of PTEN regulation are still incomplete.

Recently, we have identified a potential tumor suppressor RBM38 (as known as RNPC1) in breast cancer [19], which also exhibited a tumor suppressor functions in colorectal cancer [20], acute myeloid leukemia [21], renal cell carcinoma [22] and hepatocellular carcinoma [23]. As a target of p53 and a member of RNA-binding protein (RBP), RBM38 played a significant role in post-transcriptional regulation, mRNA splicing, surveillance, stabilization and translation in gene expression [19, 24, 25]. It functions as a tumor suppressor by regulating various genes expression via binding to the AU/U- rich elements (AREs) within their 3′-untranslated region (3′-UTR). For instance, RBM38 could increase p21, p73, Hu antigen-R (HuR) and growth differentiation factor 15 (GDF15) expressions by stabilizing their mRNAs [26–28]. Conversely, RBM38 could also decrease the stability of p63, c-Myc and mouse double minute 2 homolog (MDM2) by bounding on their mRNA to inhibit tumor cells proliferation [29–31]. In our previous studies, RBM38 was also proved to regulate the expression of ER, and progesterone receptor (PR) by stabilizing their stability to regulate the breast cancer proliferation, both in vivo and vitro [32]. To investigate more RBM38 binding genes, RNA-immunoprecipitation and sequencing (RIP-Seq), an experimental method to identify enrichment and targets of RBPs, was used [33, 34]. According to the results of RIP-seq (unpublished data), we found that PTEN mRNA might be bound by RBM38 directly in breast cancer. Therefore, we hypothesize PTEN expression can be regulated by a special mechanism in breast cancer: an RNA-binding protein, RBM38 can bind PTEN mRNA directly and affect its stability.

## Methods

### Cell culture, transfection and treatment

The human breast cancer cell line BT474 was purchased from American Type Culture Collection (ATCC, USA). MDA-MB-453 was obtained from Shanghai Institutes for Biological Sciences, Chinese Academy of Science. Cells were cultured in high glucose dulbecco’s modified eagle medium (DMEM, Wisent, China) with 10% fetal bovine serum (FBS, Gibco, USA), 1% penicillin-streptomycin (Hyclone, USA) solution at 37 °C and 5% CO_2_ incubator.

RBM38 overexpression and knockdown lentivirus constructs were obtained and generated as previously described [35]. The breast cancer cells were transfected with RBM38 overexpression lentivirus (termed as RBM38), a negative control (termed as NC), a scramble control (termed as SCR) and RBM38 knockdown lentivirus (termed as sh1, sh2).

For PTEN siRNA (GenePharma, China) transfection, BT474 and MDA-MB-453 RBM38 overexpression (RBM38) and the control (NC) cells were seeded in 6-well plates overnight and then transfected with PTEN-siRNA (si1, si2) and the control (Ctl), using Lipofectamine^®^ 3000 transfection agent (Invitrogen, USA). Experiments were performed 72 h after transfection. See Supplementary methods for sequences. For PTEN inhibitors treatment, BT474 and MDA-MB-453 cells transfected with RBM38 overexpression lentivirus were treated with the PTEN inhibitors, 0.4 μM SF1670 (Sigma, USA) or 0.3 μM Vo-ophic (Sigma, USA), for 48 h. While MDA-MB-453 cells transfected with RBM38 overexpression lentivirus were treated with the PTEN inhibitors, 1.0 μM SF1670 or 0.3 μM Vo-ophic, for 48 h. To determine PTEN mRNA stability, cells were treated with 5 μg/ml actinomyclin D (Act D) (MedChem Express, USA) for 0, 2, 4, 6, 8 h.

### Tissue samples and immunohistochemistry (IHC) staining

The breast cancer samples used in IHC were obtained from 77 patients who had received breast cancer treatment at the First Affiliated Hospital of Nanjing Medical University, China, in 2013. While the freshly isolated breast cancer tissues used in the Western blotting analysis were obtained from 48 patients who had received breast cancer treatment at the First Affiliated Hospital of Nanjing Medical University, China, in 2017. The use and collection of the samples was reviewed and approved by the Institutional Ethics Committee of the First Affiliated Hospital of Nanjing Medical University. TNM staging was defined according to the American Joint Committee on Cancer (AJCC) (the 6th version, 2002). The same tissue samples were stained with RBM38 and PTEN antibody respectively. The RBM38 antibody (LifeSpan Biosciences, USA) was used at the dilution of 1:300. The PTEN antibody (Cell Signaling technology, USA) was used at the dilution of 1:125. The rabbit polyclonal antibody was used as anti-RBM38 and PTEN primary antibody. The breast cancer tissues were scored by semiquantitative analysis upon a well-established immunoreactivity scoring system (IRS) [36]. The staining intensity (SI) was scored on a scale of 0–3. The score 0 was attained for totally negative cases. For weak, moderate, and strong staining, the scores were 1, 2 and 3. Secondly, the percentage of positive cells (PTEN) was scored into five categories: no staining, 1–10, 11–50, 51–80, 81–100 percentage positive cells. And the scores were 0, 1, 2, 3 and 4. An IRS was calculated by multiplying the percentage of PTEN times the SI score, resulting in a scale from 0 to 12. The IRS was divided into three groups: negative (IRS 0–3), or low staining (IRS 4–7) and high staining (IRS 8–12). The tissue microarrays were observed under 200 × magnifications.

### Western blotting analysis and immunoprecipitation

Western blot analysis was performed as described previously [35]. The antibodies used in this study were: anti-rabbit RBM38 (Santa Cruz, USA), anti-rabbit PTEN (Cell Signaling technology, USA), anti-mouse β-actin (Cell Signaling technology, USA). The anti-rabbit and anti-mouse antibodies were purchased from Cell Signaling technology (USA). β-actin was used to normalize protein loading. For immunoprecipitation, cell lysates were incubated with 8 μl of PTEN antibody and 50% protein A agarose beads (Thermo, USA) for 3 h. The immuncomplexes were washed and used for PTEN lipid phosphatase activity.

### RNA isolation, reverse transcription and quantitative RT-PCR (qRT-PCR)

Total RNA was isolated by Trizol reagent (TaKaRa, Japan), and cDNA was synthesized using Primescript RT Reagent (TaKaRa, Japan) according to the manufacturer’s protocols. All PCR reactions were performed using the fluorescent SYBR Green I methodology. qRT-PCR was performed on StepOnePlus Real-Time PCR system (Applied Biosystems, USA) with fast start universal SYBR Green master (Roche, Switzerland) according to the manufacturer’s instructions. The PCR primers used here were shown in the supplementary materials. The relative quantification was calculated by the 2^-ΔΔCt^ method.

### PTEN lipid phosphatase activity assay

Lipid phosphatase activity of PTEN was measured on immunoprecipitated PTEN using a PTEN activity ELISA kit (Echelon, USA), according to the manufacturer’s instructions. Briefly, the stable cells transfected with ectopic and know-down RBM38 lentivirus were plated in 6-well plates for 48 h. Next, cells were washed with cold PBS and lysed with lysis buffer which was composed of IP-lysis buffer (Thermo, USA) and protease inhibitor cocktail (Sigma, USA), then incubated for 15 min with constant agitation on ice. After transferring supernatant to a fresh cooled centrifuge, for immunoprecipitation the PTEN protein, anti-PTEN antibody (CST, USA) were added to the lysate and incubated overnight on ice, then cell lysates were pre-cleared with Protein A agarose beads (Thermo, USA) and incubated for 2 h on ice. The last products were resuspended by PTEN reaction buffer and used to measure the activity of PTEN. The PIP_2_ produced was determined, in triplicate experiments, by comparison to a standard curve consisting of PIP_2_ standards bound to the ELISA plate.

### RNA electrophoretic mobility shift assay (REMSA)

The expressed RBM38 protein were produced and purified as previously describe [32] . The UCSC Genome Browser (Http://genome.ucsc.edu/) and a two-dimensional structure prediction algorithm (RNAfold, http://rna.tbi.univie.ac.at/cgi-bin/RNAfold.cgi) were used to choose the potential ARE sites of PTEN mRNA 3′-UTR. To generate REMSA probes, various regions in PTEN and p21 3′-UTR were PCR-amplified using primers containing T7 promoter sequence (5′- TAATACGACTCACTATAGGG -3′). The sequences of PTEN PCR products were listed in Additional file 1: Table S1. The sequences of P21 PCR product was as previously describe [32]. RNA probes were made by in vitro transcription with a MEGA shortscript Kit (Ambion, USA) in the presence of biotin-16-UTP (Roche, Switzerland) following the manufacturer’s instruction.

REMSA was performed with a LightShift chemiluminescent RNA EMSA Kit (Thermo, USA) following the manufacturer’s instruction. Briefly, 4 mg/ml purified RBM38 [32], 10 mg/ml of tRNA, 2 nM biotin-labeled RNA probe were mixed in a REMSA binding buffer [10 mM HEPES (pH 7.3), 20 mM KCl, 1 mM MgCl_2_, 1 mM dithiothreitol] and incubated for 30 min at room temperature. RNA/protein complexes were then electrophoreticed by 4% native polyacrylamide gel and transferred to nylon membrane (Thermo, USA). RNA was cross-linked with a UV lamp at a distance of 0.5 cm from the membrane for 3 min. The membrane was blocked in blocking buffer for 15 min and replaced the blocking buffer with conjugate/blocking buffer. After washed with 1× wash buffer for 3 times, membrane was incubated in substrate equilibration buffer for 5 min. Then, the membrane was incubated in working solution and exposed.

### Dual-luciferase reporter assay

A dual-luciferase reporter assay was performed in triplicate according to manufacturer’s protocol (Progema, USA). BT474 and MDA-MB-453 RBM38 overexpression (RBM38) and the control (NC) cells were seeded in a 24-well plate at a concentration of 10^5^ cells per well and cultured overnight. Then, a Renilla luciferase vector (Progema, USA) and 200 ng of the pGL3 reporters containing multi regions of PTEN 3′-UTR were co-transfected into these cells. The Renilla plasmid was also transfected to normalize transfection efficiency. After 48 h post-transfection, luciferase activity was determined using the dual-Luciferase reporter assay system (Progema, USA). The relative luciferase activity of the fold change is a product of the luciferase activity of the RBM38 divided by that induced by NC.

### RNA immunoprecipitation (RIP) assay

RIP was carried out as previously described [35]. In brief, the breast cancer cells (2 × 10^7^) were lysed with RNA immunoprecipitation lysis buffer (Millipore, USA) and then incubated with 5 μg of rabbit polyclonal anti-RBM38 or non-immunized rabbit IgG at 4 °C overnight. The RNA-protein immunocomplexes were brought down by protein A/G magnetic beads, followed by RNA purification. After that, the purified RNA was subjected to RT-PCR and qRT-PCR to measure the level of PTEN, P21, HuR transcript.

### Colony formation assay

The cells used for colony were treated with PTEN inhibitor or transfected with PTEN-siRNA. For colony formation assay, the breast cancer cells were plated into 6-well plates (500 cells/ well). After 2 weeks of regular culture, the formatting colonies were fixed with methanol and stained with Giemsa (Sigma, USA) after washed by PBS twice, then dried at room temperature. The number of colonies was counted using ImageJ counting particle tools [37] and indicated as a bar graph.

### Statistical analysis

The data were analyzed using the SPSS 20.0 software. All experiments in this study were repeated in triplicate, unless otherwise specified. The correlation between RBM38 and the clinic pathological parameters was analyzed by χ^2^ test. The linear correlation analysis was used to assess the correlation between RBM38 and PTEN. Student t-test was used to analyze the differences between means of independent groups. *P* < 0.05 was considered to indicate a statistically significant difference.

## Results

### The expressions of RBM38 and PTEN was positively correlated in human breast cancer tissues

**Fig. 1:**
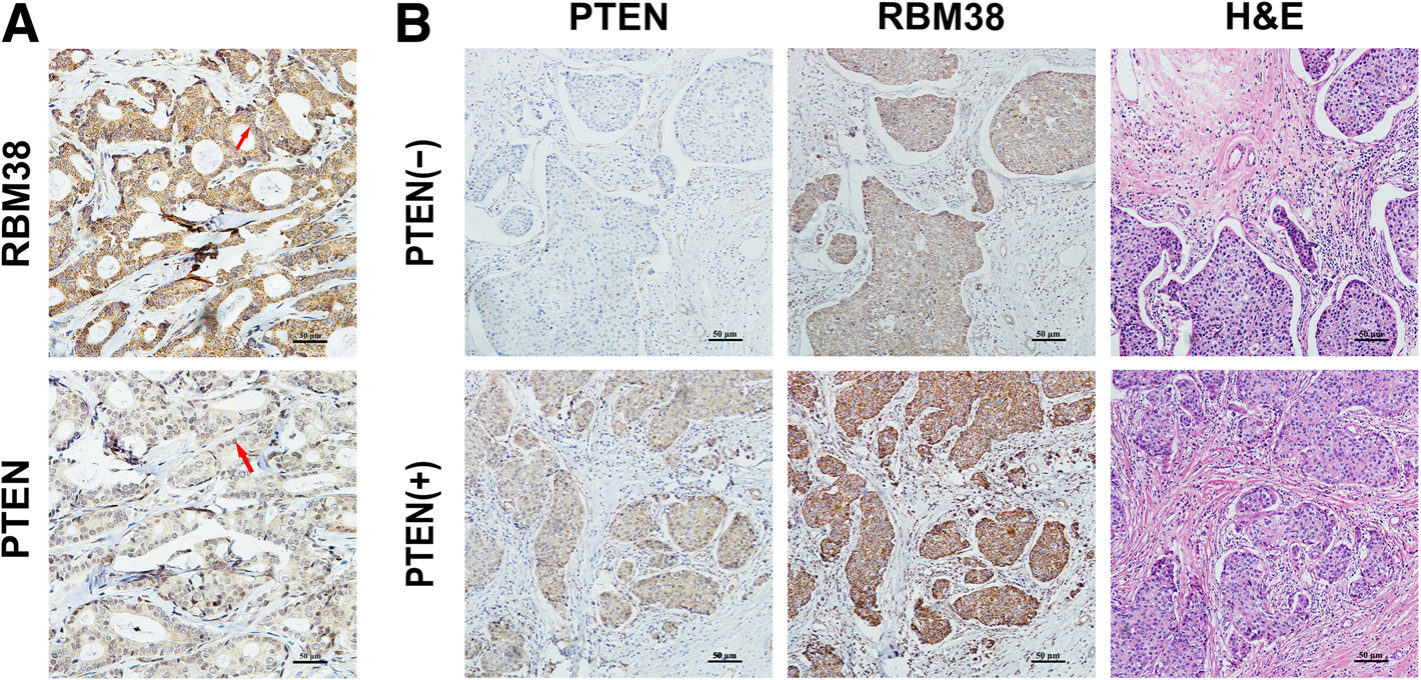
RBM38 expression was positively correlated 1 with PTEN in breast cancer tissues. **a** Immunohistochemistry analysis of PTEN and RBM38 in breast cancer tissue at 200 × magnification. RBM38 was mainly expressed in cytoplasm and PTEN was expressed in the cytoplasm and nucleus. The arrow showed the location of RBM38 and PTEN. **b** Representative images of moderate and the relative RBM38 staining in PTEN positive and negative breast cancer tissues

The protein expression of PTEN and RBM38 was measured in 77 breast cancer tissues by IHC. Both RBM38 and PTEN were mainly expressed in the cytoplasm and nucleus (Fig. 1a). The representative images of RBM38 and PTEN expression in breast cancer tissues were showed in Fig. 1b. The correlation between RBM38 expression and clinic pathological features was showed in Table 1 (*P* < 0.05). Meanwhile, we also found that there was a positive correlation between the protein expression of RBM38 and PTEN in breast cancer tissues by using Western blotting (Additional file 2: Figure S1). These results implicated that the expression PTEN and RBM38 were positively correlated.

**Table 1:** Association of RBM38 with PTEN and clinic pathological characteristics of breast cancer

Clinic pathological characteristics	RBM38 expression			
	No. of cases	Low (%)	High (%)	*P*-value
Age	0.206			
< 50	36	15(41.67)	21(58.33)	
≥ 50	41	23(56.10)	18(43.90)	
Tumor size	0.935			
≤ 2 cm	26	13(50.00)	13(50.00)	
> 2 cm	51	25(49.02)	26(50.98)	
TNM stage	0.055			
I – II	72	38(52.78)	34(47.22)	
III	5	0(0)	5(100.00)	
PTEN	0.014			
negative	46	28(60.87)	18(39.13)	
positive	31	10(32.26)	21(67.74)	

### RBM38 upregulated PTEN expression and activity in breast cancer cells

**Fig. 2:**
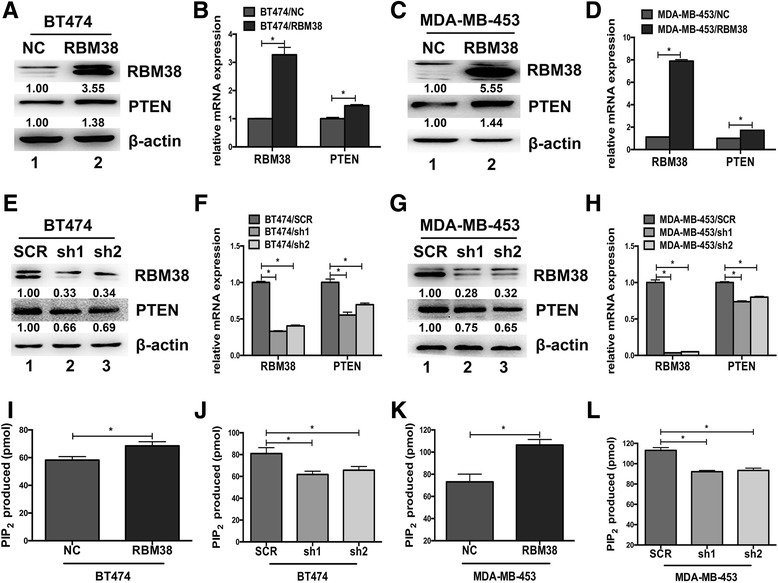
RBM38 upregulated the expression and the activity of PTEN in breast cancer cells. (**a**-**d**) BT474 and MDA-MB-453 cells were transfected with lentivirus containing either RBM38 overexpression or the control (NC). The expression of PTEN was increased after RBM38 upregulated both in protein (**a**, **c**) and mRNA levels (**b**, **d**). **e**-**h** BT474 and MDA-MB-453 cells were transfected with the knockdown RBM38 (sh1, sh2) and the control (SCR) lentivirus. PTEN expression was decreased after RBM38 downregulated both in protein (**e**, **g**) and mRNA levels (**f**, **h**). Western blot and qRT-PCR were performed to examine the expression of RBM38 and PTEN. **i**-**l** BT474 and MDA-MB-453 cells were transfected with lentivirus, containing RBM38 and control (NC), followed by PTEN immunoprecipitation (IP) and determination of its PIP3 phosphatase activity. The relative lipid phosphatase activity of PTEN was enhanced (**i**, **k**). Meanwhile, the PTEN lipid phosphatase activity in BT474 and MDA-MB-453 transfected with SCR, sh1and sh2 was inhibited (**j**, **l**). Data were means of three separate experiments and presented as mean ± SEM. Asterisk (*) indicates *P*-value <0.05. β-actin was used as protein loading control

To explore the relation between PTEN and RBM38, we constructed stable RBM38 overexpression and knockdown cell lines using two breast cancer cell lines, BT474 and MDA-MB-453. In the BT474 and MDA-MB-453 cells that were transfected with lentivirus containing RBM38 overexpression (RBM38), the control (NC), PTEN expression was apparently upregulated both in protein and mRNA levels (Fig. 2a-d). Moreover, in BT474 and MDA-MB-453 cells that were transfected with RBM38 knockdown (sh1, sh2) and the control (SCR) lentivirus, the protein and mRNA expression of PTEN was obviously decreased (Fig. 2e-h). Since the lipid phosphatase activity of PTEN is critical for its tumor suppressor function [38], we tested the PTEN activity of the stable RBM38 overexpression and knockdown cell lines. We found that the activity of PTEN could be enhanced by RBM38 in the stable RBM38 overexpression cell lines (Fig. 2i, k). Similarly, the activity of PTEN was significantly decreased in the sable RBM38 knockdown cell lines (Fig. 2j, l). These indicated that RBM38 could positively affect the expression and activity of PTEN in breast cancer cell.

### RBM38 bound to PTEN mRNA directly in breast cancer cells

**Fig. 3:**
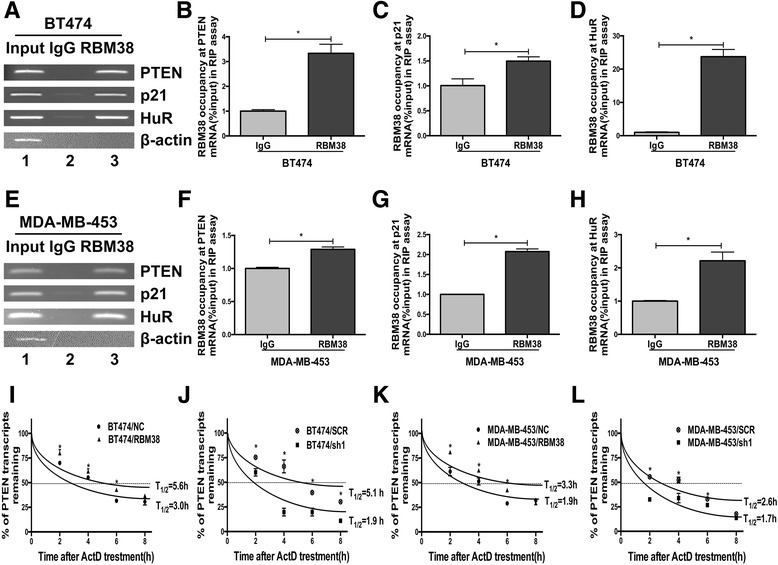
RBM38 directly bound to PTEN transcript and regulated its mRNA stability. **a**-**h** RBM38 associated with PTEN transcript in vivo. (**a**-**d**) BT474 and (**e**-**h**) MDA-MB-453 cell lysates were immunoprecipitated with RBM38 and control IgG antibodies followed by RT-PCR (**a**, **e**) and qRT-PCR (**b**-**d**, **f**-**h**), measuring transcript levels of PTEN, HuR, p21 and β-actin within RBM38 or IgG immunocompleses. **i**-**l** The half-life of PTEN transcript was up-regulated by RBM38. **a**, **b** PTEN mRNA expression in BT474 and MDA-31 MB-453 transfected with RBM38 overexpression lentivirus and the control (NC) after treatment with 5 μg/ml actinomycin D for 0 h, 2 h, 4 h, 6 h and 8 h. **c**, **d** PTEN mRNA expression in BT474 and MDA-MB-453 transfected with RBM38 knockdown lentivirus (sh1) and the negative control (SCR) after treatment with 5 μg/ml actinomycin D for 0 h, 2 h, 4 h, 6 h and 8 h. Data were means of three separate experiments and presented as mean ± SEM. * *P* < 0.05.

RNA immunoprecipitation (RIP) assay followed by RT-PCR and qRT-PCR was used to determine whether RBM38 could bind to PTEN mRNA directly in breast cancer cells. It was found that PTEN mRNA transcript was present in RBM38, but not in the control IgG immunocomplexes both in BT474 and MDA-MB-453 cells, (Fig. 3a, e). p21 and HuR mRNAs were positive controls, which were reported to form immune complexes with RBM38 [26, 28]. RBM38 was unable to bind to β-actin mRNA, a negative control. It revealed that RBM38 could bind to PTEN mRNA directly.

### RBM38 increased PTEN mRNA stability in breast cancer cells

After overexpression of RBM38, the half-life of PTEN mRNA increased from 3.0 h to 5.6 h (Fig. 3i) in BT474 cell. Similarly, in MDA-MB-453 cell, the half-life of PTEN mRNA increased from 1.9 h to 3.3 h (Fig. 3k). Accordingly, the half-life of PTEN mRNA decreased after RBM38 knockdown. In BT474 cell, the half-life of PTEN mRNA decreased from 5.1 h to 1.9 h after transfecting with sh1 (Fig. 3j). While, in MDA-MB-453 cell, the half-life of PTEN mRNA decreased from 2.6 h to 1.7 h after transfecting with sh1. These data suggested that RBM38 could directly bind to PTEN and stabilize the PTEN mRNA.

### RBM38 bound directly to AU-rich element within the PTEN 3′-UTR

**Fig. 4:**
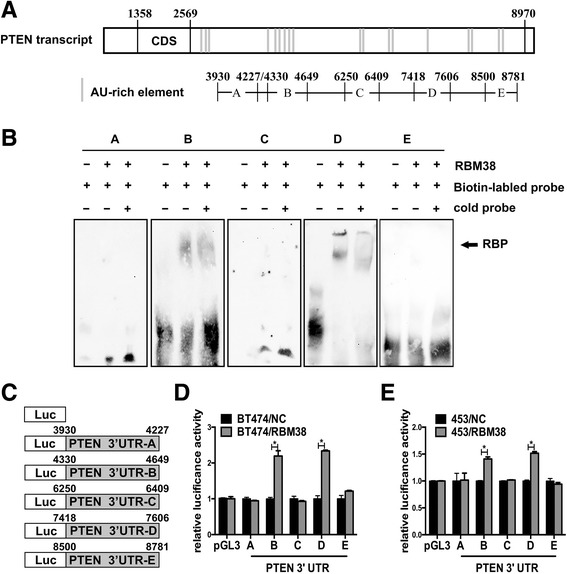
Multiple regions in the PTEN 3′-UTR were bound by RBM38 and responsive to RBM38. **a** Schematic representation of PTEN transcript and the location of probes used for REMSA. The AU-rich elements were shown in shaded boxes. **b** RBM38 bound to multiple regions in PTEN 3′-UTR, including probes B and D. RBM38 could not bind to probes **a**, **c** and **e**. **c** The schematic representation of the pGL3-PTEN 3′-UTR vector with various regions of PTEN 3′-UTR. **d**-**e** In BT474 (**d**) and MDA-MB-453 (**e**) cells, the relative luciferase activity of the reporter containing PTEN 3′-UTR B and D was increased by RBM38. The relative luciferase activity was calculated as a ratio of luciferase activity induced by RBM38 divided by that induced by an empty vector (mean ± SEM). * *P* < 0.05

REMSA was performed to demonstrate the direct binding site (s) of RBM38 in PTEN mRNA. RBM38 usually bound to the AREs in 3′-UTR of targeted genes. The UCSC Genome Browser (http://genome.ucsc.edu/) was used to analysis PTEN mRNA and provided a series of AREs in its 3′-UTR. Moreover, a two-dimensional structure prediction algorithm (RNAfold, http://rna.tbi.univie.ac.at/cgi-bin/RNAfold.cgi) was applied to support the probability of RBM38 to bind to these sites. The site B (probe B), C (probe C), D (probe D) and E (probe E) containing AU-rich elements were designed. Additionally, A (probe A) which is not rich in AU elements was chosen as the negative controls (Fig. 4a). It was found that RBM38 protein formed a complex with probe B and probe D, but not probe A, probe C and probe E (Fig. 4b). These data proved that RBM38 could bind to two ARE regions of PTEN mRNA 3′-UTR.

We performed a dual-luciferase assay in BT474 and MDA-MB-453 cells to functionally confirm the ARE regions that were required for RBM38 binding to PTEN mRNA. The luciferase activity for the reporters carrying PTEN 3′-UTR –B and D were significantly increased by RBM38. On the contrary, the PTEN 3′-UTR A, C and E were not responsive to RBM38 (Fig. 4d, e). Generally, these data suggested that PTEN 3′-UTR-B and D were responsible for RBM38 to enhance PTEN expression.

### The reduction of PTEN attenuated RBM38-mediated growth suppression

**Fig. 5:**
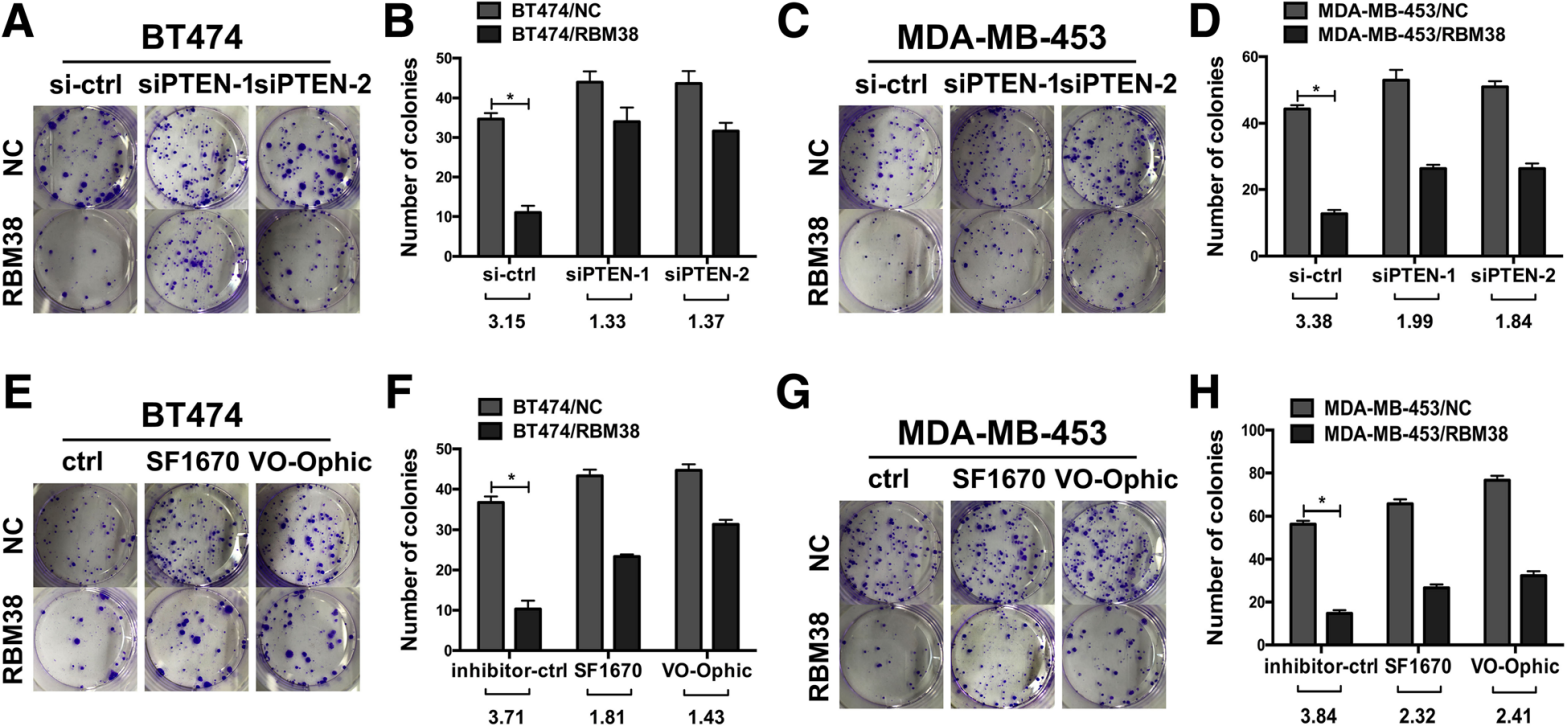
Reduction of PTEN expression suppressed the cell proliferation driven by RBM38. The effect of PTEN on RBM38-transfected cells was examined by colony formation assay. **a**-**d** BT474 and MDA-MB-453 cells transfected with RBM38 overexpression and the control (NC) lentivirus were transduced with siRNA against PTEN. The RBM38-induced growth suppression was decreased after reduction of PTEN in BT474 (**a**, **b**) and MDA-MB-453 (**c**, **d**) cells. **e**-**h** The cells transfected with RBM38 overexpression and the control (NC) lentivirus were treated with the PTEN inhibitors, SF1670 and VO-Ophic, followed by colony formation assay. The RBM38-mediated growth suppression was reduced after treatment with PTEN inhibitors in BT474 (**e**, **f**) and MDA-MB-453 (**g**, **h**) cells. The fold change was calculated as a ratio of the number of colonies induced by the control (NC) divided by that induced by the RBM38. Data were means 61 of three separate experiments and presented as mean ± SEM. * *P* < 0.05

To investigate whether the increased expression of PTEN by RBM38 affected RBM38-induced growth suppression, RBM38 stable breast cancer cells BT474 and MDA-MB-453 that transfected with RBM38 and NC lentivirus were transfected with siRNA against PTEN (siPTEN-1, siPTEN-2) and the control (ctrl), followed by a colony formation assay. The PTEN knockdown efficiency was shown in the Additional file 3: Figure S2. The colony formation assay showed that overexpression of RBM38 restrained the growth of the breast cancer cell BT474 and MDA-MB-453, which was consistent with our previous finding [19]. After transfecting with PTEN siRNA, the colony fold (NC/RBM38) was apparently decreased (Fig. 5a-d). It indicated that the reduction of PTEN expression diminished the RBM38-mediated growth suppression in BT474 and MDA-MB-453. Besides, RBM38 stable breast cancer cells BT474 and MDA-MB-453 cells that transfected with RBM38 and NC lentivirus were treated with PTEN inhibitors, SF1670 and VO-Ophic. After treating with PTEN inhibitors, the colony fold (NC/RBM38) was obviously attenuated (Fig. 5e-h). It proved that the growth suppression ability of RBM38 was decreased when PTEN expression was inhibited in BT474 and MDA-MB-453 cells. Together, these data proved that the growth suppression induced by RBM38 was obviously associated with PTEN expression.

## Discussion

In the present study, we found that RBM38, a member of RRM family of RNA-binding proteins, could enhance PTEN mRNA stability by directly targeting the ARE of PTEN mRNA 3′-UTR, and then increase the PTEN protein expression. Moreover, the cell proliferation function of RBM38 in breast cancer was significantly affected by the expression of PTEN.

According to the result of the RIP-seq, we found that PTEN expression might be bound by RBM38. Then, we confirmed that RBM38 expression was positively correlated with PTEN expression in breast cancer tissues by IHC. Moreover, overexpression of RBM38 increased PTEN mRNA and protein levels in breast cancer cells. Conversely, knockdown of RBM38 negatively affected PTEN mRNA and protein level. Considering PTEN protein is a lipid phosphatase that dephosphorylates PIP_3_ [39], it showed that PTEN activity was enhanced after overexpression of RBM38. Consistently, knockdown of RBM38 decreased the lipid phosphatase activity of PTEN. RBM38 can regulate the targeted genes mRNA stability by binding to their AREs in mRNA transcript [29, 32, 40]. Indeed, we found RBM38 overexpression was able to enhance PTEN mRNA transcript stability by prolonging its half-time after treatment with Act D for various times. Besides, RBM38 knockdown decreased the half-time of PTEN transcript obviously. RIP was used to prove that RBM38 could bind to PTEN mRNA directly. In further studies, REMSA and dual-luciferase reporter assays were used to confirm that RBM38 bound directly to the two AREs in the 3′-UTR of PTEN mRNA to enhance its mRNA and protein expression in breast cancer cells. The expression of PTEN is known to be under tight control at both the transcription and translational levels. However, the regulation of PTEN mRNA stability by RBPs is less well characterized. In this study, we have identified a novel mechanism in which RBM38 enhanced the PTEN transcript by binding directly the AREs in the 3′-UTR of PTEN mRNA to suppress PTEN protein expression.

PTEN is a well-characterized tumor suppressor in breast cancer. The expression of PTEN was found to be closely related to the phenotype, prognosis and drug selection in breast cancer [41]. Our previous study found that RBM38 acted as a tumor suppressor in breast cancer [20]. Knockdown the expression of RBM38 is sufficient to induce the proliferation, aggressive behavior, and survival of breast cancer cells both in vivo and in vitro, while opposite effects were observed when RBM38 was overexpressed [19]. Besides, we found RBM38 is a favorable factor in relapse-free survival (RFS). KM plot was shown in Additional file 4: Figure S3. Many studied found that RBM38 exhibited its tumor growth inhibiting activity by stabilization of p21, p73 and HuR transcripts or destabilization of MDM2 and c-Myc transcripts, via binding to AREs in the 3′-UTR of their mRNAs [31]. Considering that both PTEN and RBM38 are tumor suppressor genes in breast cancer, we speculated that whether RBM38 functioned as a tumor suppressor by enhancing PTEN expression. siRNA for PTEN was used to repress the expression of PTEN. Colony formation assay proved that after ectopic RBM38 breast cancer cells transfecting with PTEN siRNA RBM38-mediated growth suppression was decreased. It implied that RBM38-mediated increase in PTEN expression play a role in the RBM38-mediated tumor suppression process. Additionally, specific PTEN inhibitors, SF1670 and Vo-ophic, were used to repress the activity or of PTEN to explore weather RBM38-induced growth suppression associated with PTEN activity. Colony formation assay proved that both SF1670 and Vo-ophic alleviated RBM38-mediated growth suppression in breast cancer cells, indicating that RBM38-induced growth suppression was related to the RBM38-mediated increase in PTEN activity. Together, we concluded RBM38 acted as a tumor suppressor partially by enhancing PTEN expression.

## Conclusions

The present study revealed a new PTEN regulating mechanism that PTEN was positively regulated by RBM38 via stabilizing its transcript stability, which in turn alleviated RBM38-mediated growth suppression. This provided a new insight into the mechanism of tumorigenesis and considering the importance of PTEN and RBM38 for the breast tumor progression and gene-targeted therapy for breast cancer.

## Additional file

Additional file 1: Table S1. Sequence of REMSA probes. (DOCX 120 kb)
Additional file 2: Figure S1. The protein expressions of RBM38 and PTEN was positively correlated in breast cancer tissues. (A) RBM38 and PTEN protein expression in 48 breast cancer tissues. The relative protein expression of RBM38 and PTEN is shown below each lane by using the RBM38 band/β-actin ratio and PTEN band/β-actin ratio. The intensity of the bands was determined by using Image J. (B) A scatter plot of RBM38 and PTEN relative protein expression in the same cancer tissue (2-tailed Spearman′s correction, *R* = 0.3864, *P* < 0.05). (JPEG 798 kb)
Additional file 3: Figure S2. PTEN expression was reduced after transfected with PTEN siRNA PTEN was reduced after transfected with siRNA against PTEN (siPTEN-1 and siPTEN-2) both in protein and mRNA levels in BT474 (A-D) and MDA-MB-453 (E-H) that transfected with ectopic RBM38 and the control (NC) lentivirus. (JPEG 1055 kb)
Additional file 4: Figure S3. RBM38 is a favorable factor in relapse-free survival (RFS) The KM plot of tumor samples with detailed clinical information which were downloaded from TCGA database (https://cancergenome.nih.gov/). The mean value was used as cutoff value when investigating the role of RBM38 in survival analysis. (JPEG 132 kb)

## Abbreviations

3′-UTR: 3′-untranslated region
Act D: Actinomycin D
AREs: AU/U-rich elements
ER: Estrogen receptors
GDF15: Growth differentiation factor 15
HuR: Hu antigen-R
IHC: Immunohistochemical
MDM2: Mouse double minute 2 homolog
PR: Progesterone receptor
PTEN: phosphatase and tensin homolog deleted on chromosome 10
RBM38: RNA binding motif protein 38
RBP: RNA-binding protein
REMSA: RNA electrophoretic mobility shift assay
RIP: RNA immunoprecipitation
RIP-Seq: RNA-immunoprecipitation and sequencing
