# Supplementary material for: PTEN expression is upregulated by a RNA-binding protein RBM38 via enhancing its mRNA stability in breast cancer
Source: J Exp Clin Cancer Res. 2017 Oct 19;36:149. doi: 10.1186/s13046-017-0620-3 (PMC5649103; doi:10.1186/s13046-017-0620-3)
Supplement: Additional file 1: Table S1. — Sequence of REMSA probes. (DOCX 120 kb) [file 13046_2017_620_MOESM1_ESM.docx]

**Additional file1: TableS1. Sequence of REMSA probes**

| Probe | Sequence |
| --- | --- |
| A | TAATACGACTCACTATAGGGAAAGACATTTGATTTTTCAGTAGAAATTGTCCTACATGTGCTTTATTGATTTGCTATTGAAAGAATAGGGTTTTTTTTTTTTTTTTTTTTTTTTTTTTTAAATGTGCAGTGTTGAATCATTTCTTCATAGTGCTCCCCCGAGTTGGGACTAGGGCTTCAATTTCACTTCTTAAAAAAAATCATCATATATTTGATATGCCCAGACTGCATACGATTTTAAGCGGAGTACAACTACTATTGTAAAGCTAATGTGAAGATATTATTAAAAAGGTTTTTTTTTCCAGAAATTTGGTGTCTT |
| B | TAATACGACTCACTATAGGGAGCTGTGGTCTGACCTAGTTAATTTACAAATACAGATTGAATAGGACCTACTAGAGCAGCATTTATAGAGTTTGATGGCAAATAGATTAGGCAGAACTTCATCTAAAATATTCTTAGTAAATAATGTTGACACGTTTTCCATACCTTGTCAGTTTCATTCAACAATTTTTAAATTTTTAACAAAGCTCTTAGGATTTACACATTTATATTTAAACATTGATATATAGAGTATTGATTGATTGCTCATAAGTTAAATTGGTAAAGTTAGAGACAACTATTCTAACACCTCACCATTGAAATTTATATGCCACCTTGTCTTT |
| C | TAATACGACTCACTATAGGGTTTGTGGCTTTTGTCTATTATTCTGTACTCTGCCATCAGCATATGGAAAGCTTCATTTACTCATCATGACTTGTGCCATATAAAAATTGATATTTCGGAATAGTCTAAAGGACTTTTTGTACTTGAATTTAATCATGTTGTTTCTAATATTCTTAAAAGC |
| D | TAATACGACTCACTATAGGGAATAAAATAATCACTCATAATCCTATCACCTGGAGACATAGCCATCGTTAATATGTTAGTGACTATACAATCATGTTTTCTTCTGTATATCCATGTATATTCTTTAAAAATGAAATTTATACTGTACCTGATCTCAAAGCTTTTTAGCTTAGTATATCTGTCATGAATTTGTAGGATGTTCCATTGCAT |
| E | TAATACGACTCACTATAGGGTATCATTTACTGGCTATTTTTTTATGTACACCTTTAGGATTTTCTGCCTACTCTATCCAGTTGTCCAAATGATATCCTACATTTTACAAATGCCCTTTCAGTTTCTATTTTCTTTTTCCATTAAATTGCCCTCATGTCCTAATGTGCAGTTTGTAAGTGTGTGTGTGTGTGTCTGTGTGTGTGTGAATTTGATTTTCAAGAGTGCTAGACTTCCAATTTGAGAGATTAAATAATTTAATTCAGGCAAACATTTTTCATTGGAATTTCACAGTTCATTGTAAT |
