# Supplementary figures and images for: PTEN expression is upregulated by a RNA-binding protein RBM38 via enhancing its mRNA stability in breast cancer
Source: J Exp Clin Cancer Res. 2017 Oct 19;36:149. doi: 10.1186/s13046-017-0620-3 (PMC5649103; doi:10.1186/s13046-017-0620-3)

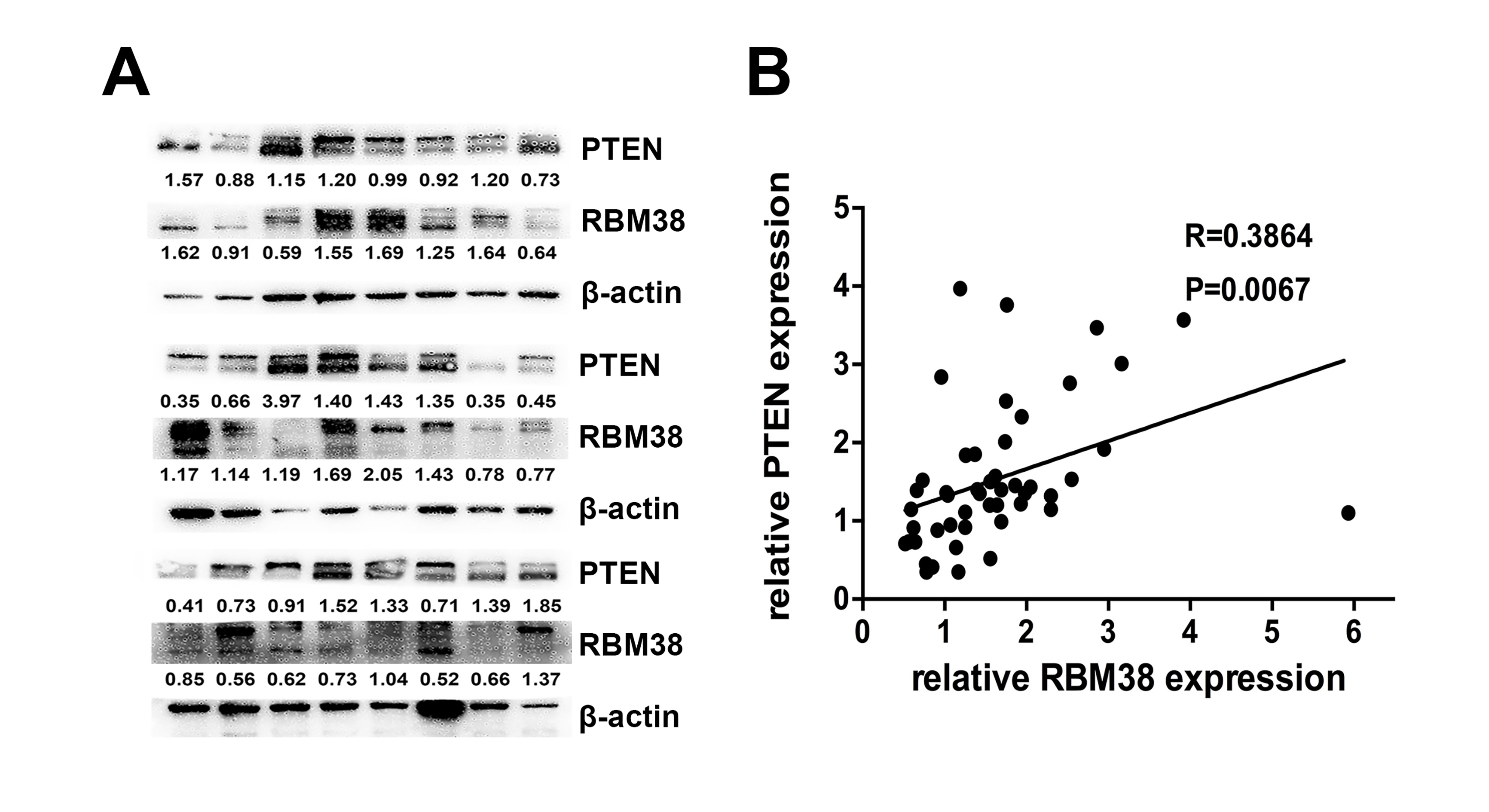

Supplement: Additional file 2: Figure S1. — The protein expressions of RBM38 and PTEN was positively correlated in breast cancer tissues. (A) RBM38 and PTEN protein expression in 48 breast cancer tissues. The relative protein expression of RBM38 and PTEN is shown below each lane by using the RBM38 band/β-actin ratio and PTEN band/β-actin ratio. The intensity of the bands was determined by using Image J. (B) A scatter plot of RBM38 and PTEN relative protein expression in the same cancer tissue (2-tailed Spearman′s correction, R = 0.3864, P < 0.05). (JPEG 798 kb) [file 13046_2017_620_MOESM2_ESM.jpg]

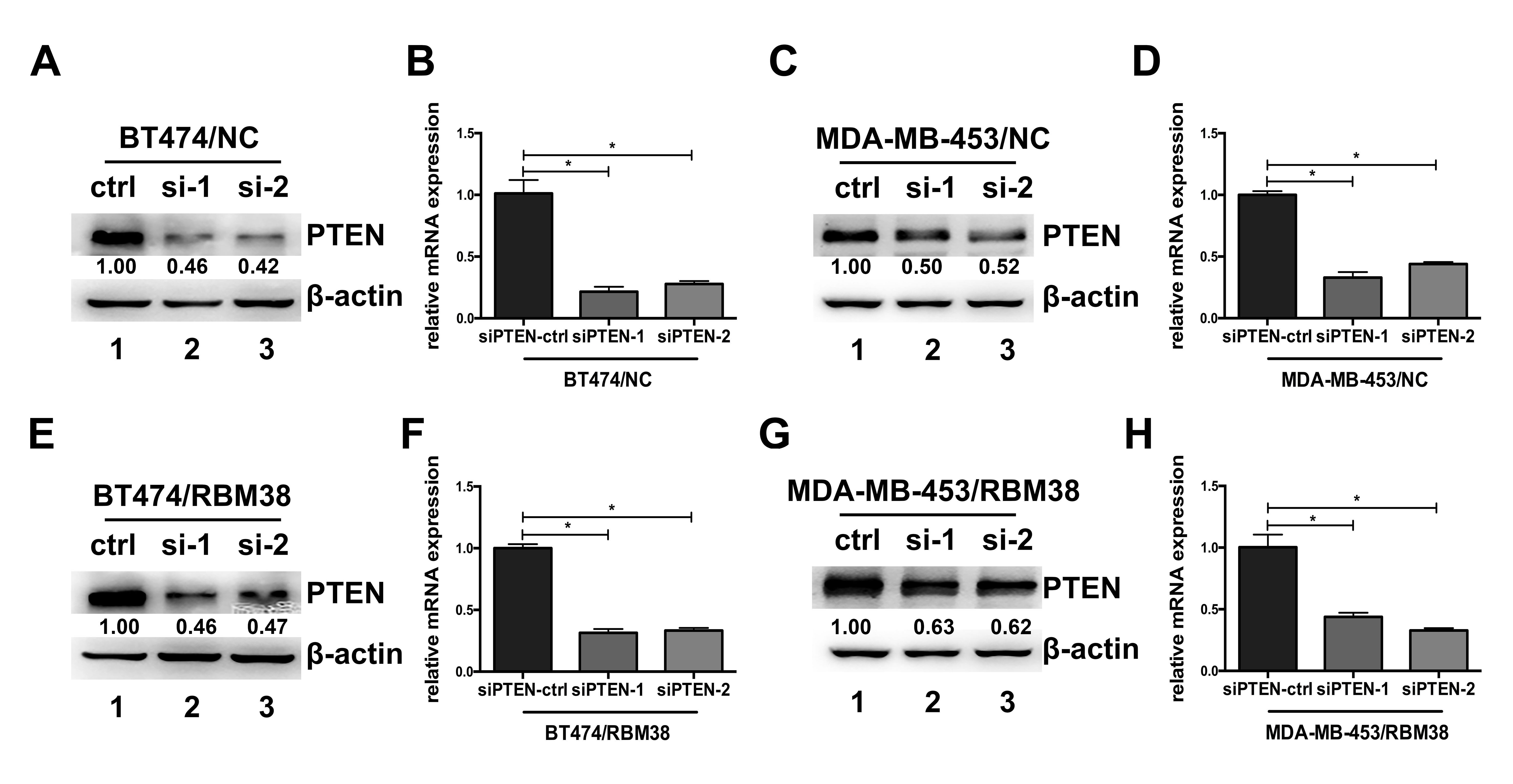

Supplement: Additional file 3: Figure S2. — PTEN expression was reduced after transfected with PTEN siRNA PTEN was reduced after transfected with siRNA against PTEN (siPTEN-1 and siPTEN-2) both in protein and mRNA levels in BT474 (A-D) and MDA-MB-453 (E-H) that transfected with ectopic RBM38 and the control (NC) lentivirus. (JPEG 1055 kb) [file 13046_2017_620_MOESM3_ESM.jpg]

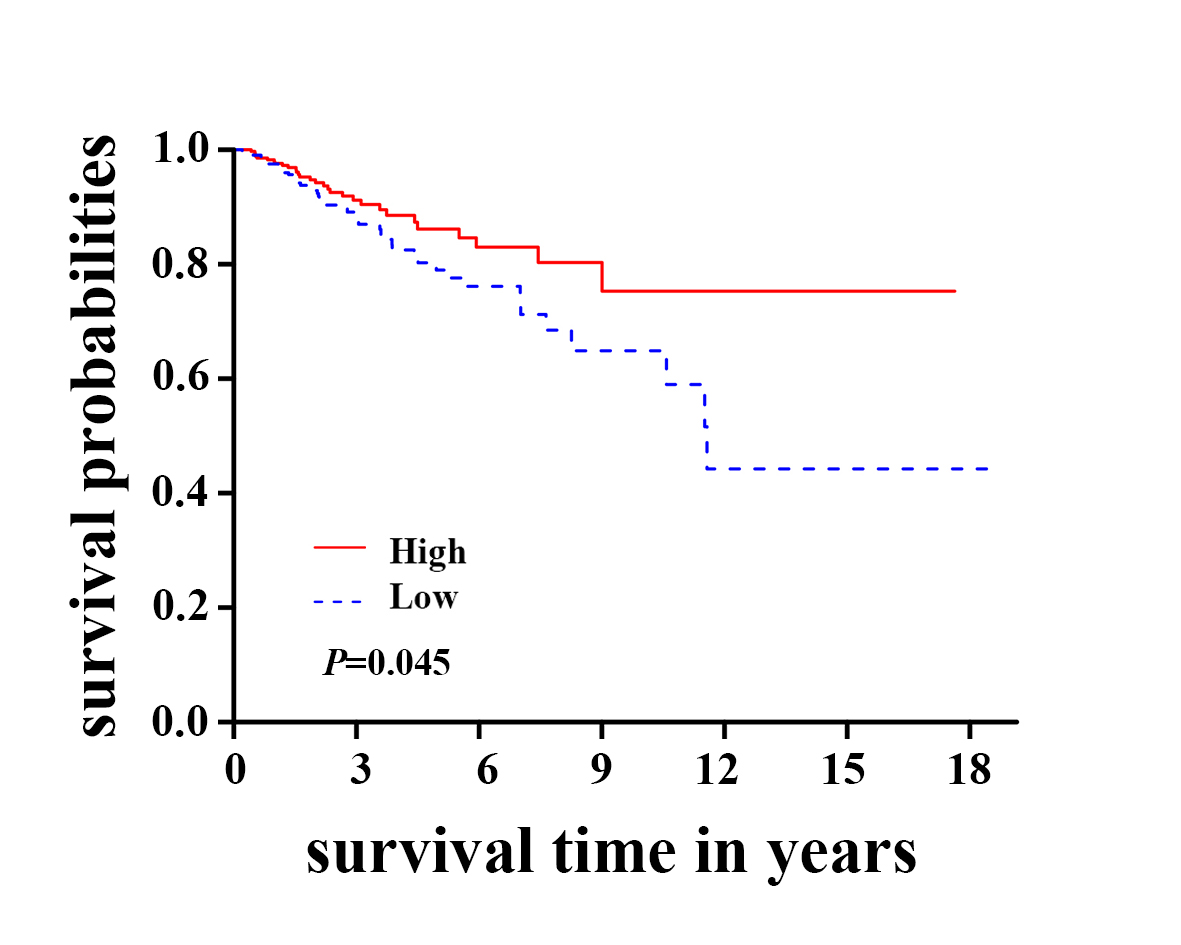

Supplement: Additional file 4: Figure S3. — RBM38 is a favorable factor in relapse-free survival (RFS) The KM plot of tumor samples with detailed clinical information which were downloaded from TCGA database (https://cancergenome.nih.gov/). The mean value was used as cutoff value when investigating the role of RBM38 in survival analysis. (JPEG 132 kb) [file 13046_2017_620_MOESM4_ESM.jpg]
